# A novel compound, SYHA1813, inhibits malignant meningioma growth directly by boosting p53 pathway activation and impairing DNA repair

**DOI:** 10.3389/fonc.2025.1522249

**Published:** 2025-02-20

**Authors:** Yanjie Lan, Shenglan Li, Jiachen Wang, Xin Yang, Can Wang, Mengqian Huang, Rong Zhang, Feng Chen, Wenbin Li

**Affiliations:** Department of Neuro-oncology, Cancer Center, Beijing Tiantan Hospital, Capital Medical University, Beijing, China

**Keywords:** meningioma, SYHA1813, therapy, p53 pathway, DNA repair

## Abstract

**Introduction:**

Meningioma is a common tumor of the central nervous system but effective therapies for malignant meningiomas are still lacking. Therefore, the development of novel therapeutic reagents is urgently needed. SYHA1813 is a novel compound and our previous study demonstrated its potent anti-tumor activity on glioblastoma through the inhibition of macrophages and human umbilical vein endothelial cells (HUVECs). However, the precise functional role of SYHA1813 in meningiomas remains unclear.

**Method:**

We aimed to investigate the direct tumor-inhibitory effects of SYHA1813 on meningioma both *in vitro* and *in vivo*, and explore its potential molecular mechanisms.

**Results:**

Our results showed that SYHA1813 suppressed the proliferation, colony formation, migration, and invasion of meningioma cells *in vitro*. Furthermore, we found SYHA1813 induced G2/M cell cycle arrest, apoptosis, and cellular senescence. Mechanistically, RNA-seq revealed that SYHA1813 activated the P53 pathway and impaired DNA repair. *In vivo*, SYHA1813 effectively inhibited the growth of meningioma xenografts in a mouse model. Additionally, in an ongoing first-inhuman phase I trial, this patient with recurrent meningioma provided preliminary clinical evidence supporting the anti-tumor activity of SYHA1813.

**Discussion:**

This study unveiled a novel antitumor mechanism of SYHA1813, showing its ability to directly target and kill meningioma cells *in vitro* and *in vivo*. Our findings highlighted the promising potential of SYHA1813 as a therapeutic agent for treating malignant meningiomas.

## Introduction

Meningiomas are the most common primary neoplasms of the central nervous system in adults, originating from the dura mater and the outer layer of the arachnoid (1, 2). Approximately 80% of meningiomas are benign and these tumors are often monitored expectantly or treated with surgery or radiotherapy, yielding favorable outcomes. The 10-year overall survival rate for benign meningiomas is estimated to be 80% to 90% (3). However, the remaining 20% of cases involve anaplastic or malignant meningiomas, which exhibit aggressive behaviors, higher recurrence rates, and worse survival outcomes (4, 5). Despite advancements in surgical resection and radiotherapy, malignant meningiomas still have a high prevalence of postoperative morbidities and radiation-induced neurotoxicity (6). As a result, drug therapy is increasingly recognized as a crucial part of meningioma treatment. A phase II trial of sunitinib, which targets vascular endothelial growth factor and platelet-derived growth factor receptors, successfully met its primary endpoint in high-grade meningiomas (7). Similarly, bevacizumab, a monoclonal antibody targeting vascular endothelial growth factor A (VEGF-A), demonstrated anti-tumor activity in a phase II trial of recurrent meningiomas (8). Furthermore, a phase II trial of pembrolizumab for recurrent meningioma achieved a partial response (9). Although several systemic agents have been tested in meningiomas, no Food and Drug Administration (FDA)-approved therapies are currently available for malignant meningiomas (10–12). Therefore, the development of novel drugs with potent antitumorigenic effects for meningiomas is urgently needed.

With increasing insights into the molecular profile of meningiomas, targeted therapies have recently garnered renewed interest in both research and clinical trials. Recent studies have shown that high expression of vascular endothelial growth factor receptor (VEGFR), platelet-derived growth factor receptor (PDEGFR) and epidermal growth factor receptor (EGFR) is associated with malignant meningiomas and shorter progression-free survival (7). Thus, targeting receptor tyrosine kinases (RTKs) has been experimentally explored as a promising approach to inhibit meningioma growth (7, 13–16). In addition, the colony-stimulating factor-1 (CSF1)/CSF1 receptor (CSF1R) axis plays a multifaceted role in the tumor microenvironment (TME), with particular relevance to its function in the survival and activation of tumor-associated macrophages (17, 18). Yeung et al. demonstrated that targeting the CSF-1/CSF-1R axis to attenuate the immunosuppressive functions of macrophages represents a promising immunotherapeutic strategy for high-grade meningiomas (19). While most previous studies focused on the therapeutic efficacy of these anti-target therapies in meningiomas through manipulating the TME, anti-VEGFR therapies were primarily developed to target tumor vasculature. However, meningioma cells also express VEGFR2, making them potential targets for VEGFR2-directed treatments (20–22). Although CSF1R-targeting therapies are beginning to shed light on how macrophage phenotypes are influenced by signaling pathways, the specific role of CSF1R-mediated signaling in meningiomas remains inadequately explored. Taken together, elucidating the direct effect of CSF1R signaling on meningioma cells could provide new insights for the development of more effective anticancer strategies for meningioma treatment.

SYHA1813 is a novel dual-target inhibitor designed and synthesized to target VEGFR and CSF1R, based on indazolylnaphthamide compounds (23). Encouragingly, ongoing clinical trials (ChiCTR2100045380) in solid cancers have demonstrated promising efficacy and safety. Our previous study reported that SYHA1813 exhibited potent preclinical antitumor effects on glioblastoma by inhibiting the viability of human umbilical vein endothelial cells (HUVECs) and macrophages (24). Additionally, it has been shown that SYHA1813 has the potential to treat B-cell lymphoma and overcome drug resistance (25). However, the functional role of SYHA1813 in meningiomas remains unclear. In the present study, we aimed to determine the tumor-inhibitory effects of SYHA1813 on malignant meningioma both *in vitro* and *in vivo*. Specifically, we investigated its ability to activate the p53 pathway and impair the DNA repair mechanism in malignant meningioma cells. Furthermore, we also explored the antitumor potential of SYHA1813 in a mouse model, with the goal of identifying a novel therapeutic agent for the effective treatment of meningiomas. Early clinical data are also presented here to support the anti-tumor activity of SYHA1813.

## Results

### SYHA1813 inhibited malignant meningioma cell growth *in vitro*

**Figure 1:**
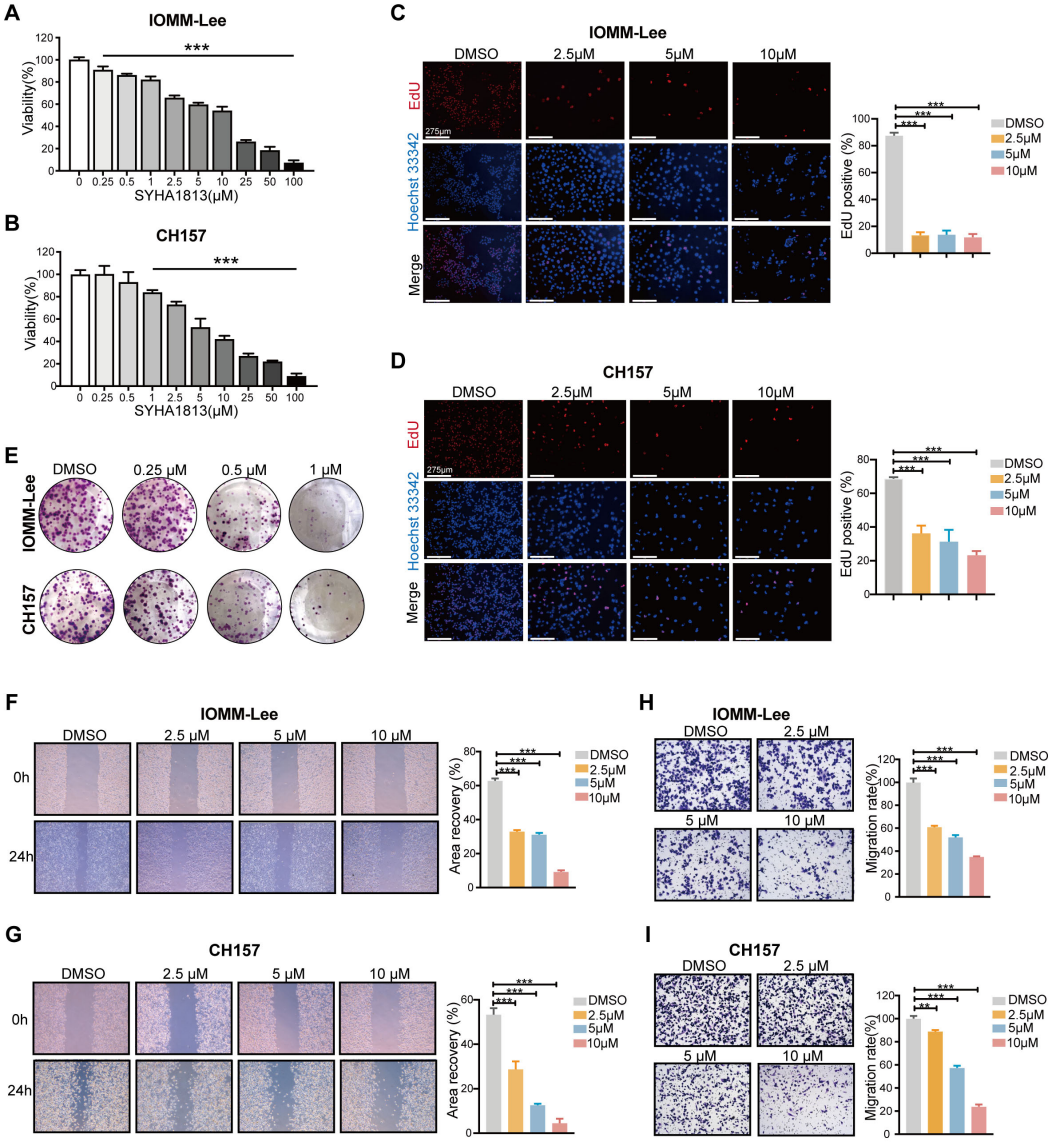
SYHA1813 exerted antitumorigenic effects on meningioma cells *in vitro*. **(A, B)** IOMM-Lee and CH157 cells were treated with different concentrations of SYHA1813 for 48h. Cell viability was measured by CCK8 assay. **(A)** IOMM-Lee; **(B)** CH157. **(C, D)** EdU staining of IOMM-Lee and CH157 cells treated with 2.5, 5, and 10μM SYHA1813 or 0.1% DMSO for 48h (EdU, red; Hoechst, blue). Representative images of EdU incorporation (left panel). Quantification of the proportion of proliferating cells (EdU^+^/Hoechst^+^ cells) in IOMM-Lee and CH157 cells (right panel). Scar bar = 275 μm. **(C)** IOMM-Lee; **(D)** CH157. **(E)** Colony formation assay for IOMM-Lee and CH157 cells was performed after treatment with 0.25, 0.5, and 1μM SYHA1813 or 0.1% DMSO. Representative images of colonies by crystal violet staining. **(F, G)** Wound healing assay was taken to detect cell migration of IOMM-Lee and CH157 cells after treatment with 2.5, 5, and 10μM SYHA1813 or 0.1% DMSO. Representative images of the wound (left panel). Quantification of the healing area (right panel). **(F)** IOMM-Lee; **(G)** CH157. **(H, I)** Transwell cell invasion assay was taken to measure cell invasion of IOMM-Lee and CH157 cells after treatment with 2.5, 5, and 10μM SYHA1813 or 0.1% DMSO. Representative images of migrated cells by crystal violet staining (left panel). Quantification of the average number of migrated cells (right panel). **(H)** IOMM-Lee; **(I)** CH157. All data shown represent the mean ± SD. Compared with the control, ***p < 0.001.

To explore the toxicity of SYHA1813 in meningioma cells, we evaluated its effect on cell growth in two human meningioma cell lines, IOMM-Lee and CH157. Both cell lines were exposed to increasing concentrations of SYHA1813 for 48 h, with the control treated with 0.1% dimethyl sulfoxide (DMSO). The CCK8 assay results showed that SYHA1813 inhibited cell viability in a dose-dependent manner in both IOMM-Lee and CH157 cells (**Figures 1A, B**). At a concentration of 2.5 μM, SYHA1813 significantly reduced cell viability in both cell lines after 48 h of treatment. Therefore, we selected doses of 2.5, 5, and 10 μM for subsequent *in vitro* studies. An 5-Ethynyl-2'-deoxyuridine (EdU) assay for detecting the effect of SYHA1813 on the proliferation of meningioma cells showed that the SYHA1813 treatment resulted in a marked decrease in the fraction of EdU-positive cells compared to the control at 48 h (**Figures 1C, D**). A colony formation assay was performed to determine whether SYHA1813 could inhibit colony formation. Notably, SYHA1813 treatment led to a reduction in the size and number of colonies compared to the control, indicating that SYHA1813 could effectively impair the clonogenic potential of IOMM-Lee and CH157 cells (**Figure 1E**). Considering that migration and invasion are key processes in meningioma progression, we further investigated the impact of SYHA1813 on these biological activities. The wound healing assay revealed that SYHA1813 remarkably inhibited the migration of IOMM-Lee and CH157 cells in a dose-dependent manner (**Figures 1F, G**). Moreover, the trans-well cell invasion assay demonstrated that SYHA1813 exposure suppressed the invasion of both cell lines (**Figures 1H, I**). These results collectively demonstrated that SYHA1813 exerts dose-dependent antitumor effects on malignant meningioma cells by inhibiting cell proliferation, migration, and invasion.

### SYHA1813 induced cell cycle arrest, apoptosis, and senescence in meningioma cells

**Figure 2:**
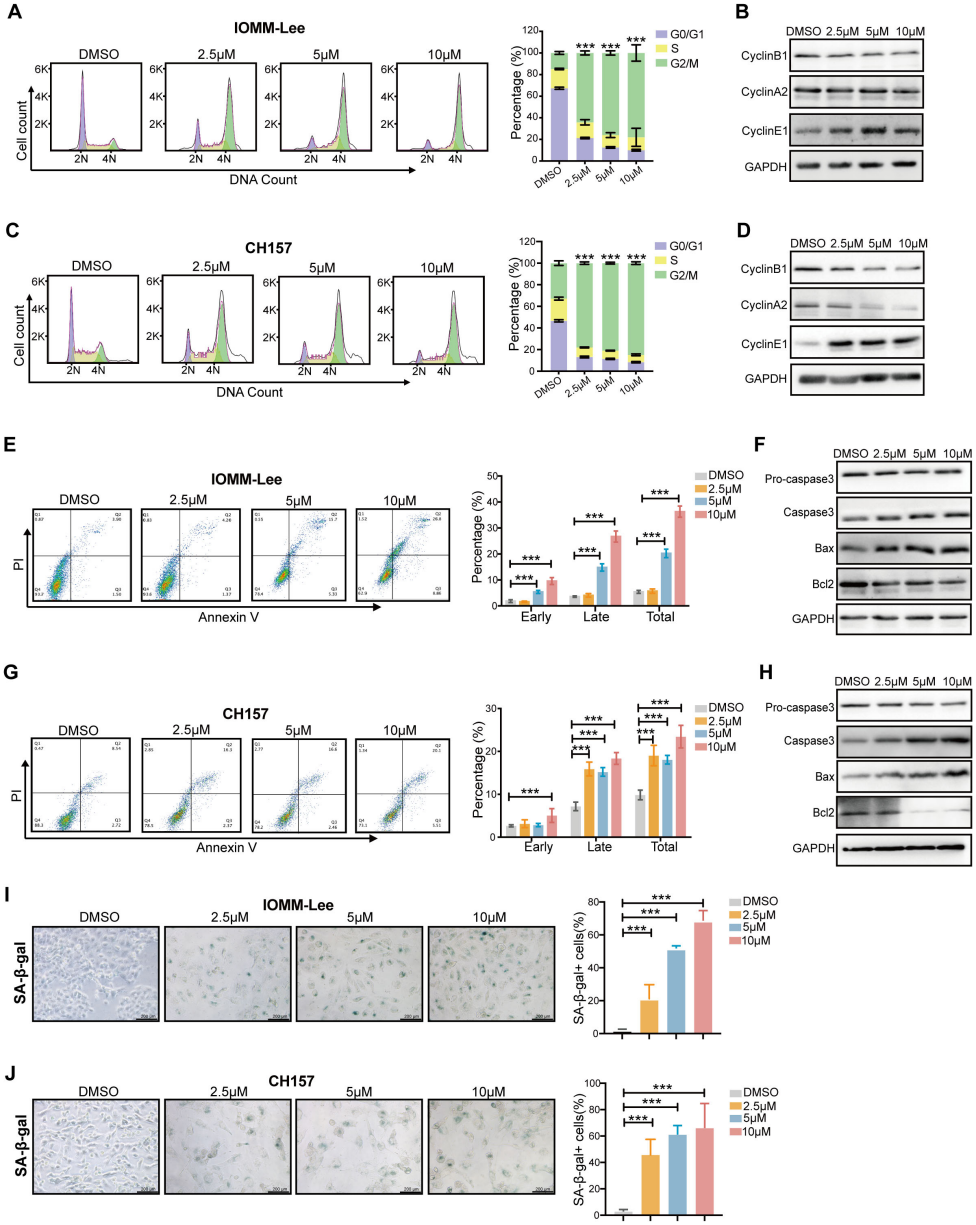
SYHA1813 arrested the cell cycle and induced cell apoptosis and senescence in meningioma cells. **(A–D)** SYAH1813 arrested cell cycle in IOMM-Lee and CH157 cells. **(A, C)** Representative flow cytometry analysis and quantitative analysis results of the cell cycle in IOMM-Lee and CH157 cells treated with 2.5, 5, and 10μM SYHA1813 or 0.1% DMSO for 48h before stained with PI; **(B, D)** Western blotting showed the protein expression of cell cycle-related proteins, Cyclin B1, Cyclin A2, and Cyclin E1, in IOMM-Lee and CH157 cells under the same treatment conditions; **(E–H)** SYAH1813 induced cell apoptosis in IOMM-Lee and CH157 cells. **(E, G)** Representative flow cytometry analysis and quantitative analysis results of the cell apoptosis in IOMM-Lee and CH157 cells treated with 2.5, 5, and 10μM SYHA1813 or 0.1% DMSO for 48h before stained with Annexin V-FITC/PI. **(F, H)** Western blotting showing the protein expression of apoptosis-related proteins, Procaspase3, Caspase3, Bax, and Bcl2, in IOMM-Lee and CH157 cells under the same treatment conditions. **(I, J)** β-galactosidase staining of IOMM-Lee and CH157 cells treated with 2.5, 5, and 10μM SYHA1813 or 0.1% DMSO for 48h. Senescent cells were identified as blue-stained cells. Representative images of senescent phenotype of cells (left panel). Quantification of the proportion of senescent cells (Blue^+^ cells/Total cells) in IOMM-Lee and CH157 cells (right panel). Scar bar =200 μm. **(I)** IOMM-Lee; **(J)** CH157. All data shown represent the mean ± SD. Compared with the control, ***p < 0.001.

To elucidate the mechanism underlying the SYHA1813-mediated inhibition of meningioma cell proliferation, we analyzed cell cycle distribution in IOMM-Lee and CH157 cells via flow cytometry following SYHA1813 treatment. The results revealed that the percentage of cells at the G2/M phase was significantly increased after SYHA1813 treatment, accompanied by the decreased percentage of cells at the G0/G1 and S phase (**Figures 2A, C**). This effect was observed to be concentration-dependent. To further validate these findings, we investigated the expression of cell cycle-related proteins by Western blot. Cyclin B1, a major checkpoint regulator of the G2/M transition, was found to be downregulated following SYHA1813 treatment. Cyclin E1 plays a critical role in the G0 to G1 phase transition and Cyclin A2 is a key regulator in the S phase. We found that the SYHA1813 concentration-dependently downregulated the protein expression of Cyclin A2 and upregulated the protein expression of Cyclin E1 (**Figures 2B, D**). Next, we assessed whether the proliferative inhibition was attributed to apoptosis. Flow cytometry analysis using Annexin V/PI staining showed that SYHA1813 induced apoptosis in both IOMM-Lee and CH157 cells in a concentration-dependent manner, with a particularly pronounced increase in late apoptotic cells (**Figures 2E, G**). Similarly, SYHA1813 treatment significantly altered the expression of apoptosis-related proteins. The levels of Caspase 3 and Bax were increased, while Bcl-2 and pro-caspase 3 were decreased (**Figures 2F, H**). Interestingly, morphological changes, including cell enlargement and flattening, were observed in IOMM-lee and CH157 cells following SYHA1813 treatment. These observations prompted us to explore whether SYAH1813 could induce cell senescence. Subsequent SA-β-Gal staining assays revealed an increase in blue-stained senescent cells, indicating that SYHA1813 treatment could promote senescence in meningioma cells (**Figures 2I, J**). Taken together, these findings suggested that SYHA1813 treatment inhibited the proliferation of IOMM-Lee and CH157 cells *in vitro* by inducing G2/M cell cycle arrest, promoting apoptosis, and triggering cellular senescence.

### SYHA1813 induced a distinct gene expression signature associated with DNA repair in meningioma cells

**Figure 3:**
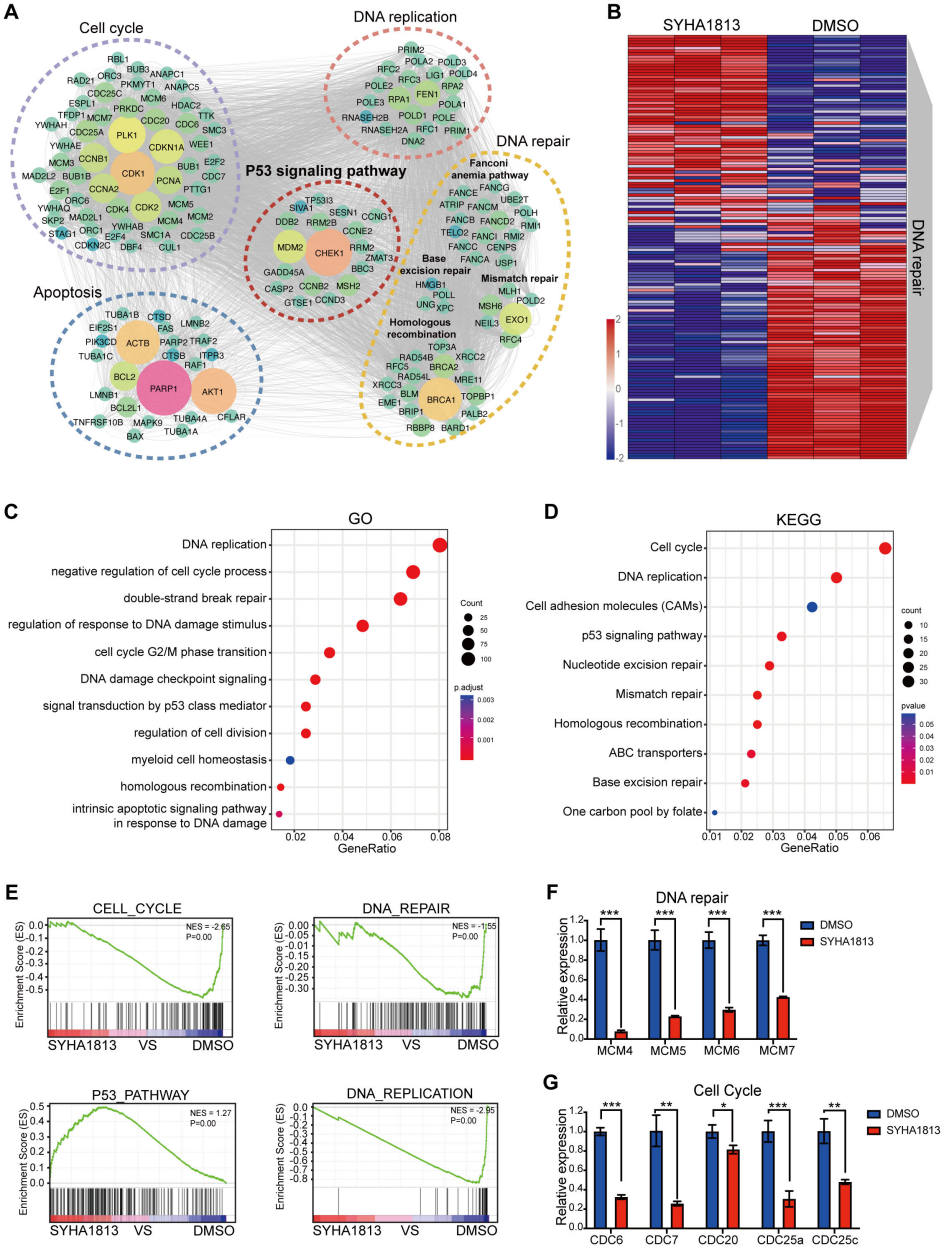
SYHA1813 altered the transcriptomics of meningioma cells. IOMM-Lee cells were treated with 5μM SYHA1813 or 0.1% DMSO for 48 h. RNA-seq analysis was used to identify the DEGs. **(A)** Gene interaction analysis showing the significantly altered expression pattern in SYHA1813-treated cells compared with control. **(B)** Heatmap showing differential expression genes related to DNA repair in SYHA1813-treated cells and control. **(C)** Gene Ontology (GO) analysis of DEGs in SYAH1813 treated cells versus control. **(D)** Kyoto Encyclopedia of Genes and Genomes (KEGG) pathway analysis of DEGs in SYAH1813-treated cells versus control. **(E)** Gene set enrichment analysis (GSEA) of genes affected in IOMM-Lee after SYHA813 treatment. **(F, G)** mRNA relative expression levels of genes related to DNA repair and cell cycle in cells treated with SYHA1813 and control. mRNA levels were normalized to the expression of GAPDH. All data shown represent the mean ± SD. Compared with the control, *p < 0.05, **p < 0.01, ***p < 0.001.

To investigate the potential molecular mechanisms underlying the effects of SYHA1813 on IOMM-Lee cell proliferation, cell cycle arrest, apoptosis, and senescence *in vitro*, transcriptome analysis was performed on IOMM-Lee cells treated with 5 μM SYHA1813 or 0.1% DMSO for 48 h. The results revealed extensive alterations in the gene expression profiles of SYHA1813-treated cells. Differentially expressed gene (DEG) analysis revealed 1,251 significantly upregulated genes and 470 significantly downregulated genes in SYHA1813-treated cells compared to controls (**Supplementary Figures S1A, B**). Enrichment analysis of these DEGs highlighted pathways associated with cell viability, such as the cell cycle, DNA repair, apoptosis, and the P53 signaling pathway (**Figure 3A**). Notably, SYHA1813 treatment resulted in a significant enrichment of genes involved in DNA repair in comparison with the controls (**Figure 3B**). Gene Ontology (GO) analyses demonstrated that SYHA1813 altered important biological processes and pathways related to DNA replication and cell cycle regulation, both of which play critical roles in modulating cell proliferation. Specifically, the DNA repair GO category revealed significant enrichment in genes associated with DNA double-strand break repair, mainly through homologous recombination (**Figure 3C**). Kyoto Encyclopedia of Genes and Genomes (KEGG) analysis revealed the activation of the P53 signaling pathway (**Figure 3D**). Consistently, gene set enrichment analysis (GSEA) showed that upregulated genes in SYHA1813-treated cells were enriched in the P53_PATHWAY, whereas genes related to the CELL_CYCLE, DNA_REPAIR, and DNA_REPLICATION pathways were significantly downregulated (**Figure 3E**). Accordingly, the mRNA expression levels of several DNA repair-associated genes, including MCM4, MCM5, MCM6, and MCM7, were decreased in SYHA1813-treated IOMM-Lee cells (**Figure 3F**). Similarly, a reduction in the expression of cell cycle-related genes, such as CDC6, CDC7, CDC20, CDC25a, and CDC25c, was observed (**Figure 3G**). These transcriptional changes align with the phenotypic alterations observed in SYHA1813-treated cells (**Figure 2**). Hence, these findings demonstrated that SYHA1813 induced a unique transcriptional signature in IOMM-Lee cells, closely linked to DNA repair and cell cycle regulation.

### The P53 pathway was involved in SYHA1813-mediated antitumorigenic effects in meningioma cells

**Figure 4:**
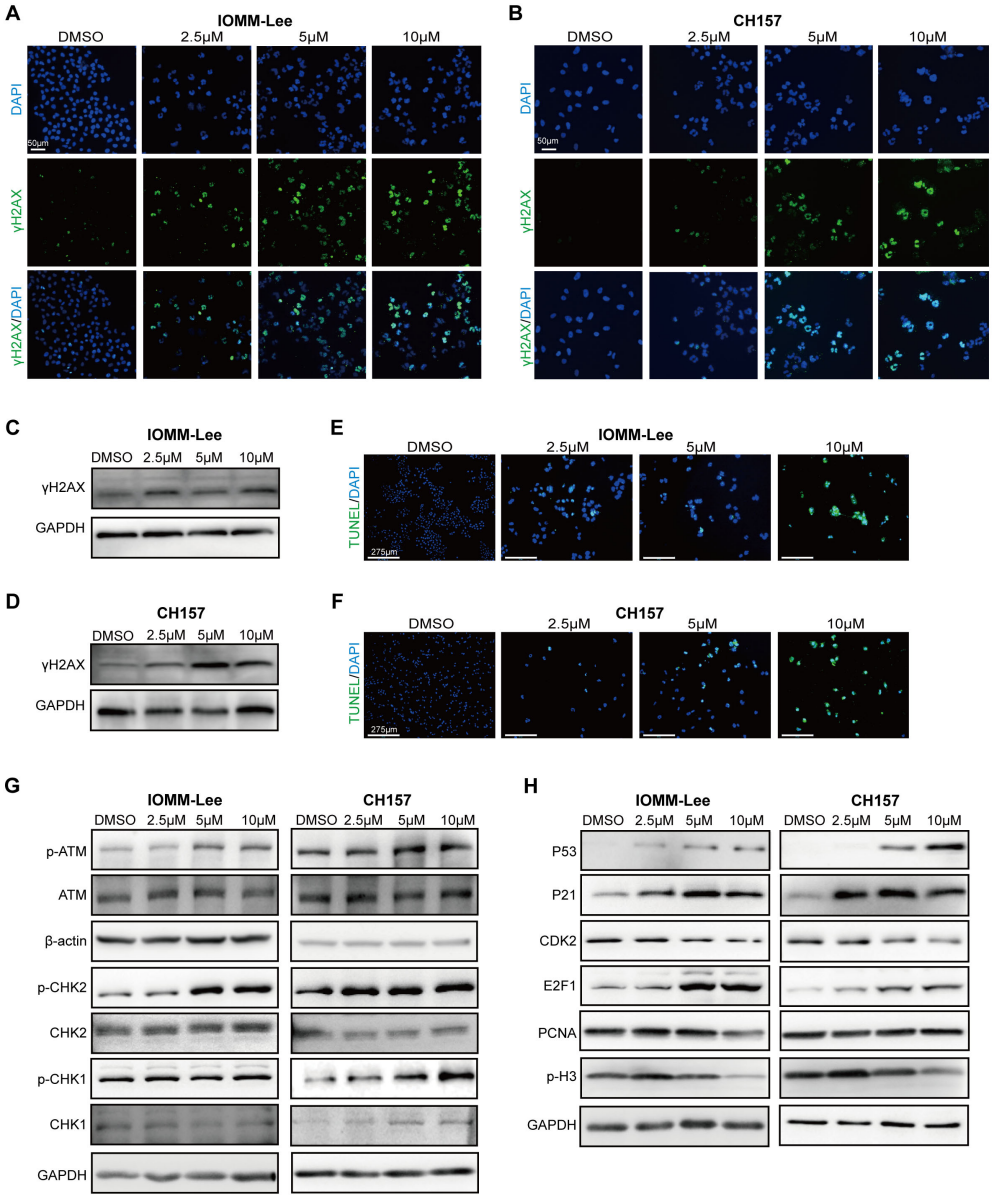
SYHA1813 activated the P53 pathway and triggered DNA damage in meningioma cells. **(A, B)** Immunofluorescence images of γ‐H2AX in IOMM-Lee and CH157 cells treated with 2.5, 5, and 10μM or 0.1% DMSO for 48h. (γH2AX, green; DAPI, blue). Scale bar = 50 μm. **(C, D)** The protein expression of γH2AX, in IOMM-Lee and CH157 cells treated with 2.5, 5, and 10μM or 0.1% DMSO for 48h. **(E, F)** TUNEL staining assay showed the distribution of TUNEL-positive cells (green color) in IOMM-Lee and CH157 cells treated with 2.5, 5, and 10μM SYHA1813 or 0.1% DMSO for 48h. DAPI staining (blue color). **(G)** Western blot analyses the protein expression of total ATM, p-ATM (Ser1981), total Chk2, p-Chk2 (Thr68), and total CHK1, p-CHK1 (Ser317) in IOMM-Lee and CH157 cells treated with 2.5, 5, and 10μM or 0.1% DMSO for 48h; GAPDH was used as a loading control. **(H)** Western blot analyses the protein involved in the P53 signal pathway and its downstream targeted effectors; GAPDH was used as a loading control.

The phosphorylated (Ser139) histone H2AX (γH2AX) is a sensitive molecular marker of DNA damage and repair. SYHA1813 treatment increased the number of γH2AX-positive cells in both IOMM-Lee and CH157 cells (**Figures 4A, B**). Meanwhile, the protein level of γH2AX was increased following SYHA1813 treatment (**Figures 4C, D**). DNA fragmentation was further confirmed by TUNEL staining. The TdT-mediated dUTP Nick-End Labeling (TUNEL) assay revealed DNA fragmentation and nuclear condensation in SYHA1813-treated IOMM-Lee and CH157 cells, with a significant increase in the rate of TUNEL-positive cells compared to controls (**Figures 4E, F**). DNA repair and damage are controlled by kinases such as ataxia telangiectasia mutated (ATM), which initiates signal transduction cascades and activates checkpoint kinase 2 (CHK2), an activator of P53 signaling (22). To further explore whether the ATM/CHK2 pathway contributes to the antitumorigenic effects of SYHA1813, we evaluated the protein levels of ATM and CHK2 in IOMM-Lee and CH157 cells. As shown in **Figure 2G**, SYHA1813 treatment increased the expression of p-ATM and p-CHK2, without affecting their total protein levels. Furthermore, SYHA1813 also increased the expression of p-CHK1 (**Figure 4G**). These findings suggested that the ATM/CHK pathway plays a significant role in SYHA1813-mediated tumor inhibition. Given the significant role of P53 signaling in tumor inhibition, cell cycle arrest, and apoptosis, we investigated the expression of key proteins in the P53 pathway and its downstream molecules. As expected, SYHA1813 treatment upregulated the protein levels of P53 and its downstream target P21 (**Figure 4H**). Additionally, SYHA1813 led to significant reductions in protein expression levels of CDK2, E2F1, PCNA, and p-H3 (**Figure 4H**), all of which are associated with cell cycle progression and enhanced DNA damage repair activity. The disruption of key signaling pathways involved in DNA damage repair represents a potential therapeutic strategy for meningioma. In summary, our results demonstrated that SYHA1813 inhibited DNA repair by activating the ATM-CHK-P53 pathway, leading to the inhibition of proliferation, cell cycle arrest, and apoptosis. These effects collectively contributed to the antitumorigenic properties of SYHA1813 in meningioma cells *in vitro*.

### SYHA1813 inhibited the tumor growth of meningioma *in vivo*

**Figure 5:**
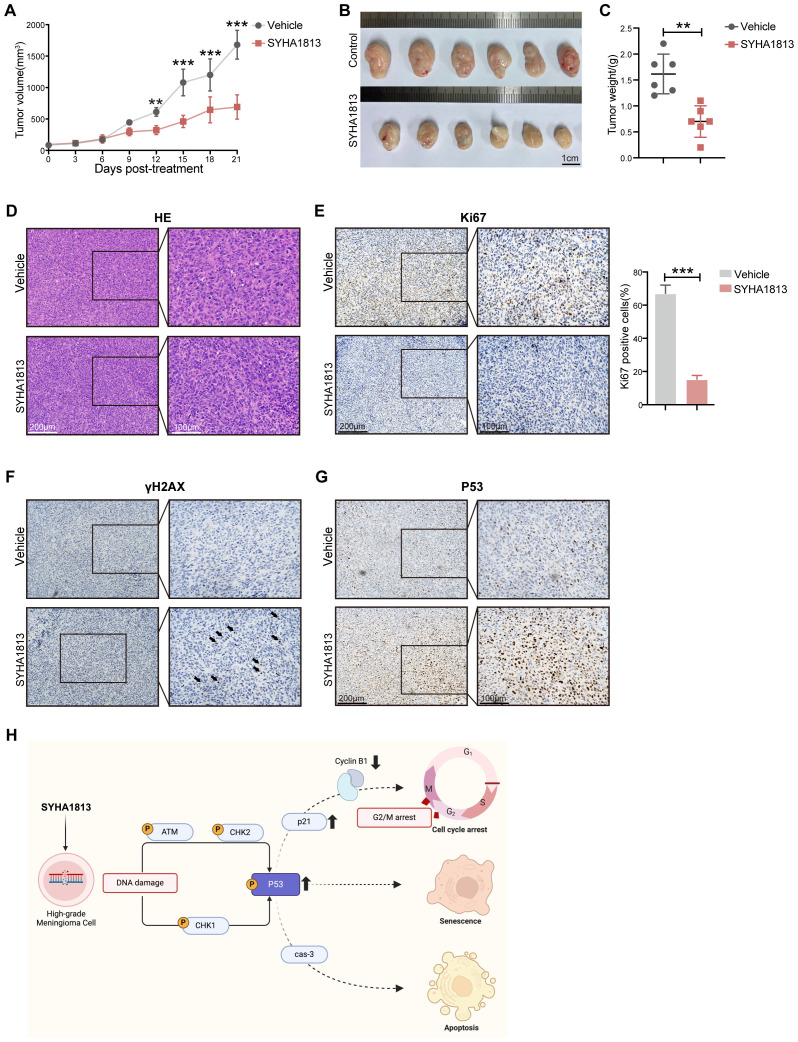
The therapeutic effect of SYAH1813 on meningioma *in vivo*. Xenograft mouse models were established with IOMM-Lee cells. At 3 days post-implantation, mice were treated with SYHA1813 at the dose of 10 mg/kg body weight or vehicle orally every day. **(A)** Tumor volume curves of mice in the vehicle and SYHA1813 treatment groups. **(B, C)** The tumors of xenograft mice were measured on the 21st day. **(B)** The resected tumor morphology image of mice treated with SYHA1813 or vehicle; **(C)** the weights of tumors. **(D)** Hematoxylin and eosin (H&E) staining in tumor sections of mice treated with SYHA1813 or vehicle. **(E)** Immunohistochemical staining for Ki-67 in tumor sections of mice treated with SYHA1813 or vehicle. Representative images of Ki-67-stained tumor sections (right panel). Quantification of the percentage of Ki67 positive cells (left panel). **(F, G)** Immunohistochemical staining for γH2AX, and P53 in tumor sections of mice treated with SYHA1813 or vehicle. **(H)** Schematic diagram of SYHA1813 exerting antitumorigenic effects in meningioma cells. SYHA1813 suppressed meningioma cells proliferation and induced cell cycle arrest, cell apoptosis and senescence, and inhibited meningioma cells growth *in vivo*. Mechanistically, SYHA1813 activated p53 via ATM/CHK signal pathway in meningioma cells. SYHA1813 further hampered DNA damage repair in meningioma cells. All data shown represent the mean ± SD. Compared with the control, **p < 0.01, ***p < 0.001.

To further evaluate the antitumor effects of SYHA1813 *in vivo*, a subcutaneous xenograft mouse model was established using IOMM-Lee cells inoculated into BALB/c nude mice. Three days after injection, the mice were randomly divided into the SYHA1813 treatment and control groups. The mice in the SYHA1813 treatment group received oral administration of SYHA1813 at a dose of 10 mg/kg body weight once daily for 21 days, while the mice in the control group received the vehicle (**Supplementary Figure S2A**). Tumor volumes effectively decreased in the SYHA813 treatment group compared to the controls (**Figure 5A**). No significant difference in body weight was observed between the two groups (**Supplementary Figure S2B**). Histopathological examination revealed no notable liver or kidney damage in the SYHA1813-treated mice (**Supplementary Figure S2C**). After 21 days of treatment, both tumor volume and weight of the SYHA1813-treated mice were significantly reduced compared to the vehicle-treated mice (**Figures 5B, C**), indicating the potent inhibitory effects of SYHA1813 on tumor growth *in vivo*. H&E staining showed that tumors in both the SYHA1813-treated and control groups exhibited features consistent with grade III meningioma (**Figure 5D**). To further investigate the anticancer effects of SYHA1813 *in vivo*, tumor cell proliferation was assessed by immunohistochemistry (IHC). The SYHA1813-treated group displayed a significantly lower percentage of Ki67-positive cells compared to the control group (**Figure 5E**), showing reduced tumor cell proliferation. Additionally, γ-H2AX staining revealed increased DNA damage and reduced proliferation of meningioma cells in tumors from SYHA1813-treated mice (**Figure 5F**). P53 staining showed that P53 protein expression levels were also elevated in tumors from the SYHA1813-treated group (**Figure 5G**). Collectively, these findings were consistent with the *in vitro* results and suggested that SYHA1813 inhibited meningioma cell growth directly by activating P53 signaling and hampering DNA damage repair (**Figure 5H**).

### Proof-of-concept clinical activity of SYHA1813 in meningioma

**Figure 6:**
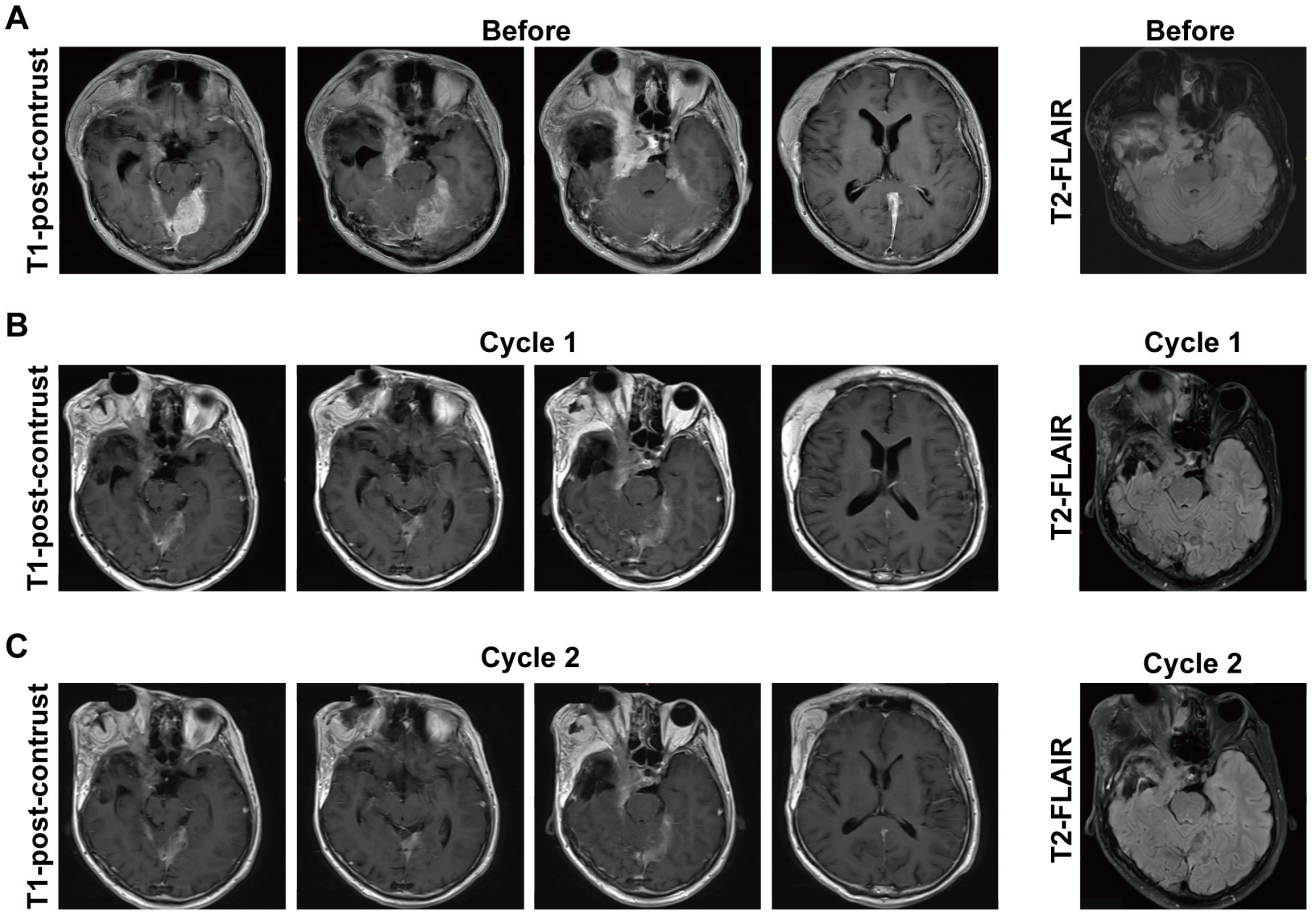
Response to SYHA1813 in meningioma patients. **(A)** Images of a patient with recurrent atypical meningioma (WHO grade II) at the screening phase, before treatment. The patient continues in the study. **(B, C)** Post-treatment images of the patient with recurrent atypical meningioma (WHO grade II) demonstrated the tumor volumes reduced after treatment. **(B)** 4-week (cycle 1) treatment of SYHA1813; **(C)** 8-week (cycle 2) treatment of SYHA1813.

SYHA1813 is currently being evaluated in an ongoing phase I dose-expansion clinical trial (ChiCTR2100045380) in patients with recurrent or advanced solid cancers, including recurrent meningioma. The study was approved by the Ethics Committee of Beijing Tiantan Hospital, Capital Medical University (approval number: YW2020-053-02). All the enrolled patients had previously undergone either gross total resection or subtotal resection and had received prior systemic therapy. Here, we provide brain magnetic resonance imaging (MRI) data from a patient with recurrent atypical meningioma [World Health Organization (WHO) grade II] during the screening phase of the trial (**Figure 6A**). This patient received oral administration of SYHA1813 at a dose of 20 mg once daily. After one cycle of SYHA1813 treatment, MRI revealed significant changes in the tumor. On T1 post-contrast enhancement imaging, reductions in tumor burden were observed (**Figure 6B**), meeting the criteria for stable disease (SD). Similarly, changes were evident in T2-weighted and fluid-attenuated inversion recovery (FLAIR) images. Following 2 cycles of SYHA1813 treatment, the patient reported significant improvements in clinical symptoms, including the alleviation of headache and facial swelling. Post-contrast T1-weighted and FLAIR imaging demonstrated further tumor response, maintaining SD status (**Figure 6C**). Notably, no adverse events were observed during the trial for this patient. Taken together, this case provided preliminary clinical evidence supporting the anti-tumor activity of SYHA1813, consistent with its *in vitro* and *in vivo* efficacy. These findings highlighted the potential of SYHA1813 as a therapeutic agent for meningioma, supporting further investigation in ongoing and future clinical trials.

## Discussion

Meningiomas are the most common primary intracranial tumor in adults (26). For high-grade meningiomas, the standard treatments include surgical resection and radiotherapy (6, 27). However, these tumors frequently recur, exhibit aggressive growth, and pose significant treatment challenges (28). In this study, we demonstrated for the first time that SYHA1813 could effectively inhibit the growth of malignant meningioma cells *in vitro*, and induce G2/M cell cycle arrest and cell apoptosis and senescence. In the meningioma xenograft models, oral administration of SYHA1813 caused tumor shrinking. Mechanistically, we demonstrated that SYHA1813 could activate the P53 signaling pathway and impair DNA damage repair via the ATM/CHK/P53 signaling pathway, contributing to its antitumor effects. These findings suggested a novel antitumorigenic mechanism for SYHA1813, involving direct tumor cell killing in meningioma. This study highlighted the preclinical potential of SYHA1813 and supported its future development for clinical applications in meningioma treatment.

The tumor suppressor P53 is a key protein implicated in numerous cellular signaling pathways, coordinating DNA repair with cell cycle arrest, senescence, and apoptosis. It plays a key role in determining cell fate between survival and death (29). IHC studies on tissue microarrays have shown that P53 over-expression negatively affects the phenotypes of meningiomas (30). In addition, defects in the P53/P21 DNA damage-signaling pathway are reported in most meningiomas (31). Thus, reactivating the P53 pathway represents a rational therapeutic strategy for meningiomas (32). Interestingly, the RNA-seq analysis in this study revealed that SYHA1813 could upregulate the expression of P53 and its downstream effector P21 in meningioma cells. This suggested that the P53 signaling pathway played a direct role in the antitumor activity of SYHA1813. To further identify the functional status of P53/P21 and their upstream regulators, we observed that SYHA1813 increased the phosphorylation of ATM, CHK2, and CHK1, suggesting activation of the P53 pathway through the ATM/CHK signaling cascade. Consistently, our *in vitro* results demonstrated that SYHA1813 could induce cell cycle arrest, apoptosis, and senescence in meningioma cells. Moreover, SYHA1813 treatment modulated the expression of apoptosis- and cell cycle-related p53 target genes, including BCL2, BAX, and Cyclin B1. This suggested that SYHA1813 might activate P53 by influencing the network that regulates the selection of p53 target genes. Meningiomas frequently exhibit activation of multiple signaling pathways, including VEGFR and CSF1R (33). Therefore, new targeted drug strategies in aggressive or recurrent meningiomas require an additional understanding of the complex signaling pathways regulated by VEGFR and CSF1R.

It has been reported that inducing non-reversible G2/M arrest may be beneficial for managing meningioma as the G2/M checkpoint plays a vital role in the survival of meningioma cells, making it an essential target for therapeutic intervention (34, 35). Interestingly, our study demonstrated that SYHA1813 treatment could induce G2/M arrest in meningioma cells and lead to an accumulation of γH2AX, a marker of DNA damage. Histone H2AX plays a crucial role in rapid phosphorylation during chromatin modification, facilitating the recruitment of other DNA damage response (DDR) proteins (36). The DDR signaling cascade phosphorylates p53, subsequently increasing the transcriptional and translational levels of downstream genes involved in cell cycle arrest, apoptosis, or senescence (37). Radiotherapy is a cornerstone of meningioma treatment (38), exerting its therapeutic effects primarily through the induction of DNA damage (39). To optimize therapeutic efficacy while minimizing toxicity, radiotherapy is often combined with chemotherapeutic agents (32, 40–42). Remarkably, SYHA1813 was found to suppress DNA damage repair and induce cell cycle arrest, suggesting its potential to enhance the radiosensitivity of meningioma cells. This observation provided a novel perspective for therapeutic strategies in meningioma patients, warranting further research into the combination of SYHA1818 with radiation therapy.

Despite the promising anti-tumor activity of SYHA1813 observed both *in vitro* and *in vivo*, this study has several limitations. First, while we demonstrated that SYHA1813 activated the P53 pathway and directly inhibited meningioma cells through the ATM/CHK2/P53 signaling pathway, P53 is a master regulator involved in some signaling pathways. It is possible that SYHA1813 might also modulate other pathways contributing to its antitumor effects, such as those regulating invasion, migration, and senescence. Thus, further studies are needed to elucidate these additional mechanisms. Second, the relationship between p53 and DNA damage repair genes was not fully addressed in the present study. A deeper investigation into the mechanisms by which SYHA1813 regulates DNA damage repair genes is necessary. Third, due to the lack of an available mouse meningioma cell line, our *in vivo* experiments were conducted using immunodeficient mice models. Developing better animal models that incorporate cell-immune microenvironment interactions will be crucial for improving the accuracy of anti-tumor activity assessments of SYHA1813 in meningioma.

In conclusion, this study demonstrated that SYHA1813 exerted potent antitumor effects both *in vitro* and *in vivo*, significantly inhibiting cell proliferation, arresting the cell cycle, and inducing apoptosis and senescence in meningioma cells. Furthermore, SYHA1813 was shown to induce DNA damage and subsequently activate the ATM/CHK/P53 signaling pathway, contributing to its antitumor effects. These findings suggest that SYHA1813 may represent a novel and effective treatment for meningioma, deserving further investigation in both preclinical and clinical settings.

## Methods

### Regents

SYHA1813 was designed and synthesized by our group. The purity of SYHA1813 was > 99%. For *in vitro* studies, SYHA1813 was prepared as 100 mmol/mL stock solutions in dimethyl sulfoxide (DMSO) and diluted to a specific concentration in the relevant assay medium. For *in vivo* studies, SYHA1813 was prepared as a self-microemulsion matrix and diluted to 10mg/kg for oral administration. The administration volume was calculated based on the body weight of each mouse (0.1 mL/10 g-body weight) before administration.

### Cell culture

IOMM-Lee cells were purchased from the American Type Culture Collection (ATCC). CH157 cells were generously given by Prof. Mei (Nanchang University). Both cells were cultured in Dulbecco’s modified Eagle’s medium (DMEM; Gibco, Thermo Fisher Scientific, USA) supplemented with 10% fetal bovine serum (FBS; Gibco, Thermo Fisher Scientific, USA) and 1% penicillin and streptomycin (Gibco, Thermo Fisher Scientific, USA), and incubated at 37°C with 5% CO_2_.

### Cell viability

IOMM-Lee and CH157 cells were seeded in each well of 96-well plates (Costar, USA) with 3-5 × 10^3^ cells/well. After 6-8 h, cells were treated with different concentrations of SYHA1813 or 0.1% DMSO, and incubated for another 48 h. A cell viability assay was performed using the Cell Counting Kit-8 (CCK-8; Dojindo, Japan) according to the manufacturer’s protocol. After 48h, 10 μl CCK-8 was added to each well. Then, cells were incubated at 37°C with 5% CO_2_ for 2h. Absorbance (A) was measured at 450 nm by a the Tecan Spark Microplate Reader (Tecan Group AG, Switzerland) to assess cell viability. OD values were calculated as cell viability (%) = [A (SYHA1813) –A (Blank)]/[A (DMSO) – A (Blank)] × 100%.

### Colony formation assay

IOMM-Lee and CH157 cells were seeded in 6-well plates (Costar, USA) at a density of 500 cells/well. After 6-8 h for cell adhesion, cells were treated with different concentrations of SYHA1813 (0.25, 0.5, and 1 μM) or 0.1% DMSO. During a cultivation period of 12 days, refreshed medium with a specific concentration of SYHA1813 periodically. Following that, cells were washed once with phosphate buffer solution (PBS; Biosharp, China), fixed with 4% paraformaldehyde for 20 min, washed again with PBS three times, and stained with 0.1% crystal violet solution (Beyotime, China) for 20 min. The stained colonies were photographed and counted.

### 5-Ethynyl-20-deoxyuridine assay

Cell proliferation was detected using a 5-ethynyl-20-deoxyuridine (EdU) assay kit (Solarbio, Beijing, China) according to the manufacturer’s instructions. Cells were seeded into 24-well plates with a density of 2 × 10^4^ cells/well. After 6 to 8 h for cell adhesion, cells were treated with different concentrations of SYHA1813 (2.5, 5, and 10 μM) or 0.1% DMSO. After 48h, cells were washed with PBS and incubated with 50 μM EdU-containing buffer for 2 h at 37°C, followed by fixing with 4% formaldehyde and permeabilizing by 0.5% Triton X-100. EdU working solution was added to the cells and incubated in the dark for 30 min. Hoechst33342 was employed for nuclear staining and the cells were then visualized under a fluorescence microscope. Fluorescence cells were calculated by ImageJ.

### Wound healing assay

IOMM-Lee or CH157 (8×10^5^ cells/well) cells were seeded in 6-well plates. Until the confluence reached 90%, a straight gap was scratched on the cell monolayer using a p200 pipette tip. Then, the cell monolayer was washed with PBS and incubated with medium without FBS, containing different concentrations of SYHA1813 (2.5, 5 and 10 μM) or 0.1% DMSO. The gap was photographed at the same position at 24 h. The distance that the cells had migrated was calculated by ImageJ.

### Transwell cell invasion assay

Transwell invasion assays were performed to evaluate the migratory effect of SYHA1813 on IOMM-Lee and CH157 cells by Transwell chamber with an 8 μm pore size polycarbonate membrane in 24‐well plates (Costar, USA). The upper Transwell chamber was coated with 60 µl of a 10 mg/ml Collagen type I solution. The cells (2×10^5^/100 μl) were then seeded into the upper Transwell chamber with serum-free medium containing different concentrations of SYHA1813 (2.5, 5, and 10 μM) or 0.1% DMSO, and the bottom wells were supplemented with 500 μL DMEM medium containing 20% FBS. After 12h incubation, invading cells on the upper layer were fixed in 4% paraformaldehyde and stained with 0.1% crystal violet solution. The cells were photographed and counted in five randomly selected fields under microscope.

### Cell cycle analysis

IOMM-Lee and CH157 cells were seeded in 6-well plates at a density of 5 × 10^5^ cells/well. The next day, the cells were treated with different concentrations of SYHA1813 (2.5, 5, and 10 μM) or 0.1% DMSO for 48 h. Cell cycle assay was performed according to the Cell Cycle and Apoptosis Analysis Kit (Beyotime, Beijing, China) manufacturers’ protocol. Cells were harvested and fixed in 70% ethanol at -4°C for 30 min and then stained with propidium iodide (PI) solution. Cell cycle phases were determined by flow cytometry.

### Apoptosis assay

IOMM-Lee and CH157 cells were seeded in 6-well plates at a density of 5 × 10^5^ cells/well. The next day, cells were treated with different concentrations of SYHA1813 (2.5, 5, and 10 μM) or 0.1% DMSO for 48 h. The apoptosis assay was performed according to the Annexin V-FITC/PI Apoptosis Kit’s (Multi Science, China) manufacturer’s instructions. In brief, cells were collected and resuspended in 500 μl binding buffer and then incubated with 10 μl PI and 5 μl Annexin V-FITC for 5 min at room temperature. Apoptotic cells were analyzed by a flow cytometer.

### SA-β-Gal staining

IOMM-Lee and CH157 cells were seeded in 24-well plates at a density of 2×10^4^ cells/ml. After 6 to 8 h for cell adhesion, cells were treated with different concentrations of SYHA1813 (2.5, 5, and 10 μM) or 0.1% DMSO. The senescence-associated β-galactosidase (SA-β-gal) stain was performed using a Cellular Senescence Assay Kit (Beyotime, China) according to the manufacturer’s instructions. Briefly, cells were fixed with fix buffer at room temperature for 15 min. They were then washed with PBS and stained with the SA-β-Gal staining working solution at 37°C for 1 day. Images were taken under a microscope on a bright field. Senescent cells were identified as blue-stained cells.

### RNA‐seq

To obtain RNA for sequencing, 2 × 10^5^ cells were seeded into 6-well plates in three replicates and treated with 5μM SYHA1813 or 0.1% DMSO for 48h at 37°C with 5% CO_2_. Total RNA was extracted using Trizol reagent (Thermo Fisher Scientific, US) according to the manufacturer’s protocol. Transcriptome sequencing was performed on the Illumina NovaSeq 6000 platform to a total target depth of 10 million 150 bp paired-end reads. Differential expression analysis was performed by the DESeq2 R package (1.16.1). Raw data have been deposited in the National Center for Biotechnology Information (NCBI) under the BioProject number PRJNA202388.

### Western blot

IOMM-Lee and CH157 cells were treated with different concentrations of SYHA1813 (2.5, 5, and 10 μM) or 0.1% DMSO for 48 h. Cells were washed with cold PBS and lysed with RIPA lysis buffer containing 1% protease inhibitor (Solarbio, China) and 1% phosphatase inhibitor (Solarbio, China). Protein was extracted for Western blotting. Total protein was resolved via sodium dodecyl sulfate-polyacrylamide gel electrophoresis and transferred to a polyvinylidene difluoride membrane. The cells were then blocked with 5% fat-free milk or Bovine serum albumin (BSA) for 1h at room temperature and the membranes were probed with primary antibodies overnight and incubated with secondary antibody. Immunoreactive bands were visualized using an enhanced chemiluminescence detection system.

### Reverse transcription -PCR

Total RNA was extracted from cells treated with 5 μM SYHA18135 or 0.1% DMSO for 48h using an RNA-Quick Purification Kit (Esunbio, China). 1 μg total RNA was reverse transcribed to cDNA by using the EasyScript^®^ All-in-One First-Strand cDNA Synthesis SuperMix for qPCR (TransGen, China). The qRT‐PCR was performed with PowerUp™ SYBR™ Green Premix (Applied Biosystems). Glyceraldehyde-3-phosphate dehydrogenase (GAPDH) was used as an internal reference. The sequences of primers are listed in **Table 1**

**Table 1:** The primers used for RT-PCR.

	Forward	Reverse
CDC20	GTTCGGGTAGCAGAACACCA	CCCCTTGATGCTGGGTGAAT
CDC25A	CGTCGTGAAGGCGCTATTTG	GCAGAGTTCTGCCTCTGTGT
CDC25C	GAGGAACCCCAAAACGTTGC	TCTATGGCCACGGTCCAAAC
CDC6	AACACAGCTGTCCCAGATCG	CACAATCATGGGGCCCTTCT
CDC7	TCAGTGCCTAACAGTGGCTG	GCTTTTGTGGTGGACTGCTG
MCM4	TCTCGTTGACAAGGTCCAGC	GCGCATTGAGCTGACAGATG
MCM5	TTGCCTACTGCCGAGTGAAG	GTGCTCCGGGTATTTCTGCT
MCM6	GCTCCTGTGAACGGGATCAA	GGAGCCTTTCAATCCAGCCT
MCM7	CCCTCGTAGTATCACGGTGC	AGCCCCAGACTCATCATCCT
GAPDH	TTAAAAGCAGCCCTGGTGAC	GACAGTCAGCCGCATCTTCT

### Immunofluorescence

γ-H2AX and 4′,6-diamidino-2-phenylindole (DAPI) staining was performed using a DNA Damage Assay Kit (Beyotime, China). IOMM-Lee and CH157 cells were treated with different doses of SYHA813 for 48h, fixed in 4% paraformaldehyde for 15 min at room temperature, and permeabilized with 0.1% Triton X-100 in PBS for 20 min. The cells were then incubated with anti-phosho-H2AX (Ser139) antibody overnight at 4^◦^C. AlexaFluor 488-conjugated anti-mouse IgG antibody was incubated for 1h at room temperature. The slides were mounted in mounting medium with DAPI before imaging. Fluorescent images were captured using the fluorescence microscope.

### TUNEL staining assay

IOMM-Lee and CH157 cells were seeded in 24-well plates at a density of 2×10^4^ cells/ml. After 6 to 8 h for cell adhesion, the cells were treated with different concentrations of SYHA1813 (2.5, 5, and 10 μM) or 0.1% DMSO. After 48h, a one-step TUNEL apoptosis assay kit (Beyotime, China) was used following the manufacturer’s protocol to detect TUNEL-positive cells (green fluorescence).

### *In vivo* xenograft mouse model

All the mice were raised under specific pathogen-free conditions in the animal facility of Tiantan Hospital, Capital Medical University. IOMM-Lee cells were prepared at a density of 1×10^7^ cells/ml in PBS. Before cell implantation, the injection sites on the mice were disinfected with 75% alcohol. 100 µL cell suspension was subcutaneously implanted into the flanks of 4-week-old female BALB/c mice. After 3 days of implantation, the mice were randomly divided into two groups. SYHA1813 with 10mg/kg of body weight or vehicle was orally administrated daily. Tumor volumes were recorded every other day with a digital caliper and were calculated based on the longest (L) and the perpendicular (S) diameters of the tumor, using the formula: Volume (mm^3^) = (L×S^2^)×1/2. Mouse body weight was also recorded every other day. After 3 weeks of treatment, the excised tumors were weighed and photographed. All mouse experiments were randomized and blinded, following the policies of the animal ethics committee of Capital Medical University (202202004).

### Hematoxylin and eosin staining

Tumor tissues were harvested from the control and SYHA1813 treatment groups, fixed in 4% paraformaldehyde for 24h, embedded in paraffin, and 5-μm sections subjected to hematoxylin and eosin (H&E). Hematoxylin staining was performed for 5 min, hydrochloric acid alcohol was used for separation, ammonia was used to bring the color back to blue, and the eosin staining solution was used for 2 min. Sections were dehydrated with graded alcohol and mounted with neutral gum.

### Immunohistochemistry

IHC staining was carried out following the manufacturer’s instructions. Tumor tissues were formalin-fixed and paraffin-embedded. Deparaffinized and stained with primary antibodies, including anti-Ki67, P53, and γ-H2.AX, overnight. Signals were visualized using DAB. Nuclei were counterstained using hematoxylin.

### Clinical trial design and conduct in phase I clinical trial

This is a multicenter open-label phase I trial to study the safety of SYHA1813 in recurrent high-grade meningioma patients (ChiCTR2100045380). The trial was conducted in Beijing Tiantan Hospital, Capital Medical University. This study was conducted in compliance with the Declaration of Helsinki, the International Council for Harmonisation Guidelines for Clinical Practice, and applicable local regulatory requirements. The instructions were approved by the Ethics Committee of Beijing Tiantan Hospital, Capital Medical University (approval number: YW2020-053-02). All patients provided written informed consent. Patients were recruited in the dose-expansion stage and cohort expansion stage. These patients were treated with different dose levels of SYHA1813 (15, 20, and 25 mg) orally once daily. The whole process included a screening period (0 to 28 days), a treatment period (3 weeks per cycle), and a follow-up period. Adverse events were monitored throughout the trial. Dose adjustments were allowed based on the occurrence of adverse events. The patients in the present study underwent 20 mg SYHA1813 once-daily treatment for a period of 21 days as a cycle if unacceptable toxicity or another discontinuation criterion was not met.

### Ethics statement

The phase I clinical trial was conducted under clinical instructions approved by the Ethics Committee of Beijing Tiantan Hospital, Capital Medical University. The approval number is YW2020-053-02). We obtained written informed consent from all participants per the principles established by the Helsinki Declaration. All mouse experiments were randomized and blinded, following the policies of the animal ethics committee of Capital Medical University. The approval number is BNI202307010.

### Statistical analysis

The statistical analyses were performed using GraphPad Prism v.8.0. Data are presented as mean ± standard deviation. Student’s t-test was used to analyze the statistical significance of differences between the two groups. Two-way ANOVA was performed for the comparison among multigroup. *P < 0.05, **P<0.01, and ***P<0.001 indicated statistically significant differences.

## Data Availability

The data presented in the study are deposited in the National Center for Biotechnology Information (NCBI) under the BioProject repository, accession number PRJNA202388.
