# Supplementary material for: A novel compound, SYHA1813, inhibits malignant meningioma growth directly by boosting p53 pathway activation and impairing DNA repair
Source: Front Oncol. 2025 Feb 20;15:1522249. doi: 10.3389/fonc.2025.1522249 (PMC11882425; doi:10.3389/fonc.2025.1522249)
Supplement: Supplementary file 1 [file DataSheet1.docx]

Supplementary Data for Yanjie Lan et al.: ***A Novel Compound, SYHA1813, Inhibits Malignant Meningioma Growth Directly by Boosting P53 Pathway Activation and Impairing DNA Repair*** (Including 2 Supplementary Figures)


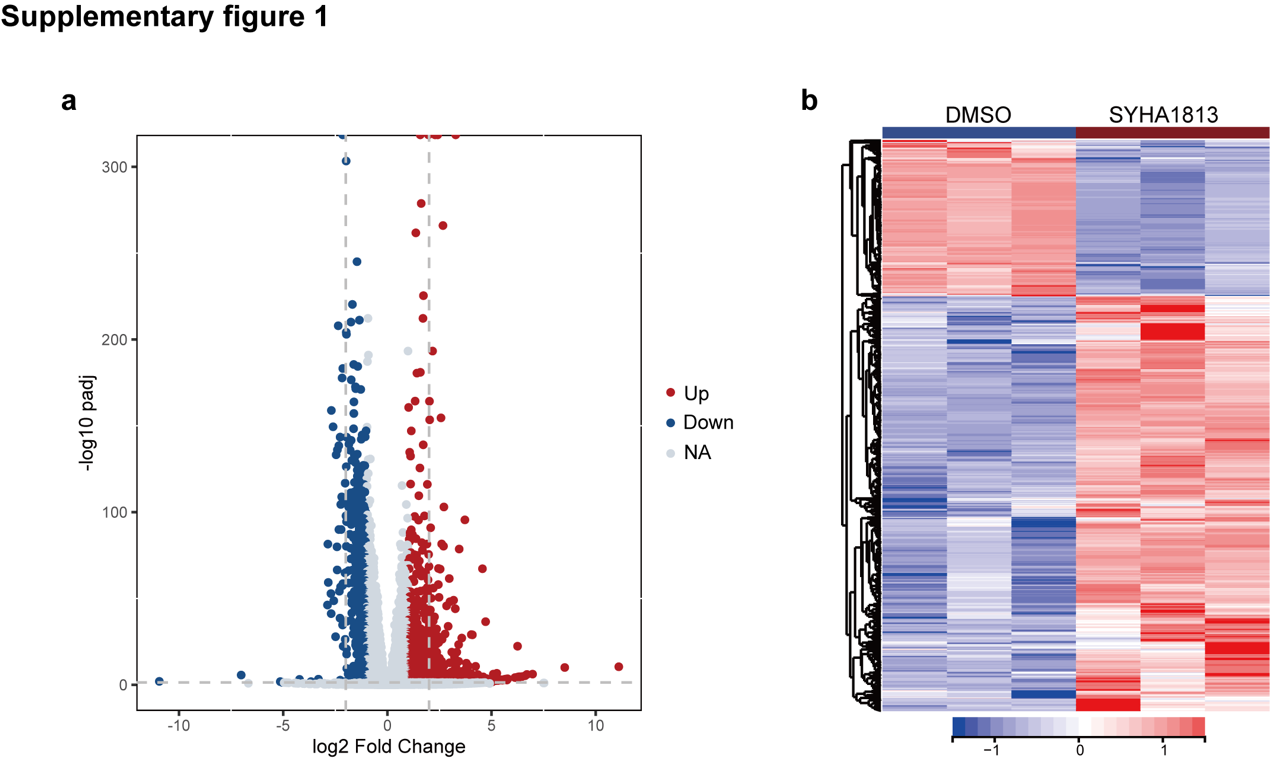


***Figure S1.*** ***SYHA1813 treatment changed gene expression profile in meningioma cells.***

***(a)*** *Volcano plot showing differentially expressed genes in SYHA1813 treated IOMM-lee cells compared to DMSO-treated controls, which is termed the total gene signature. 470 downregulated and 1251 upregulated genes were differentially expressed in SYHA1813 treated IOMM-Lee cells.* ***(b)*** *Heatmap showing differential expression genes in in* *SYHA1813 treated cells and control. |Log2foldchange | > 1, p value <0.05 (n = 3).*


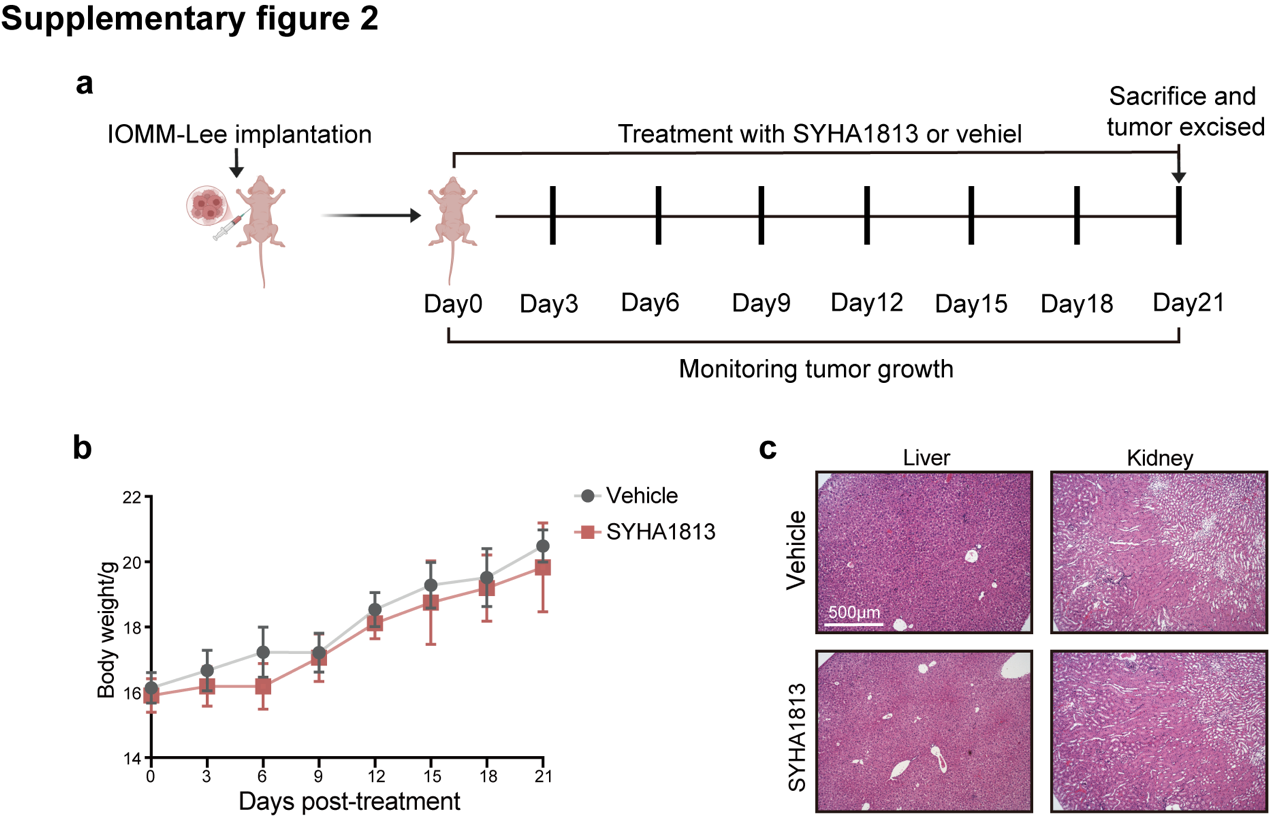


**F*igure S2.*** ***SYHA1813 treatment promised safety in meningioma xenograft mouse model.***

*(a)Schematic diagram of the established protocol of the mice models and treatment in SYHA1813 and vehicle group. (b) Body wight of mice in SYHA1813 treated group and vehicle group. (c) Immunohistochemical staining for hematoxylin and eosin (H&E) staining in liver and kidney from mice treated with SYHA1813 or vehicle.*
